# Ion channels and cardiac disease: mechanisms and functions of a disintegrin and metalloproteases and their substrates

**DOI:** 10.3389/fcell.2026.1856816

**Published:** 2026-07-31

**Authors:** Yaru Liu, Linhui Xia, Xiangyu Wang, Wenxuan Wang, Huizhu Yang, Xinjie Wang, Haoran Ding, Yiran Li, Shuai Luo, Joseph Adu-Amankwaah, Jinxiang Yuan, Rubin Tan, Kai Meng, Jianping Zhu

**Affiliations:** 1 College of Life Sciences, Shandong Normal University, Jinan, China; 2 College of Clinical Medical, Jining Medical University, Jining, China; 3 College of Stomatology, Jining Medical University, Jining, China; 4 College of Medical Imaging and Laboratory, Jining Medical University, Jining, China; 5 Shandong Lifei Biological Group, Qingdao, China; 6 Department of Pharmacology, School of Medicine, Southern University of Science and Technology, Shenzhen, Guangdong, China; 7 Lin He’s Academician Workstation of New Medicine and Clinical Translation, Jining Medical University, Jining, China; 8 Department of Physiology, School of Basic Medical Sciences, Southwest Medical University, Luzhou, China; 9 Department of Cardiology, Institute of Cardiovascular Research, The Affiliated Hospital, Southwest Medical University, Luzhou, China

**Keywords:** ADAM, calcium overload, cardiac ion channel, heart disease, mitochondrial function

## Abstract

Normal physiological activity of the heart depends on the coordinated regulation of various ion channels in cardiomyocytes (CM), and dysfunction of these channels serves as a key pathological basis for cardiac diseases, including arrhythmias and heart failure Mitochondria play a central role in maintaining the cardiac redox balance and calcium (Ca^2+^) homeostasis, and cardiac mitochondrial dysfunction constitutes a core mechanism underlying ion channel dysregulation and heart disease pathogenesis. A disintegrin and metalloproteinases (ADAMs) are a family of transmembrane proteases that play critical roles in cell adhesion, migration, and intercellular communication *via* the cleavage of diverse substrates on the plasma membrane. The abnormal expression of various bioactive ADAM family substrates and their hydrolysis are closely related to the occurrence of many cardiac diseases. Previous studies have shown that ADAMs and their substrates participate in the maintenance of cardiac Ca^2+^ homeostasis, action potential formation, and electrical signal transduction by affecting the expression, localization, and function of cardiac ion channels. This review systematically summarizes the regulatory effects of ADAMs and their substrates on cardiac ion channels and discusses their potential relationship with mitochondrial dysfunction and cardiac remodeling. We also summarize the current therapeutic drugs targeting cardiac ion channels, to provide potential strategies for mechanistic research and clinical interventions for heart disease.

## 1 Introduction

The heart acts as a contractile pump that is primarily responsible for moving blood throughout the body, delivering nutrients to organs, and removing metabolic waste (Miller et al., 2014). The contractile capability of the heart is contingent upon its normal electrophysiological activity, which is chiefly governed by the coordinated actions of various ion channels (Varró et al., 2021).

Ion channels in the heart are mainly divided into excitable and non-excitable ion channels. Cardiac excitable ion channels mainly include voltage-gated sodium channels (VGSCs), Voltage-gated calcium channels (VGCCs), and voltage-gated and inwardly rectifying potassium (K^+^) channels (Alsaloum et al., 2025). The inward sodium (Na^+^) and Ca^2+^ currents together with the outward K^+^ currents generated by activation of these ion channels determine the dynamic changes in the myocardial membrane potential and play important roles in different stages of myocardial action potential (AP) formation and conduction (Garcia-Elias and Benito, 2018; Varró et al., 2021; Yan et al., 2023; Liu et al., 2025). AP generation induces membrane depolarization and initiates cardiac excitation-contraction coupling, which increases the intracellular Ca^2+^ concentration by opening VGCCs on the membrane and RYR receptors on the sarcoplasmic reticulum (SR) (Blatter et al., 2021). Genetic mutations, pathological conditions, and pharmacological agents can modify the cardiac function, thereby affecting the function or density of cardiac ion channels. This process, referred to as cardiac electrophysiological remodeling, affects cardiac repolarization and may lead to arrhythmias, heart failure (HF), cardiomyopathy, myocardial infarction (MI), cardiac ion channel diseases, and other cardiac pathologies (Varró et al., 2021). A disintegrin and metalloproteinases (ADAMs) are Zn^2+^-dependent transmembrane metalloproteinases belonging to the adamalysin superfamily (Sharma and Singh, 2023). Under physiological conditions, ADAMs are essential for intercellular communication by mediating cell adhesion and cleaving the extracellular domains of growth factors, receptors, cytokines, and adhesion molecules on the cell membrane (Giebeler and Zigrino, 2016; Kawai et al., 2021). Additionally, ADAMs are vital to embryonic development by regulating fundamental processes, such as cell proliferation and differentiation, which are critical for tissue and organ formation during early development (Guo et al., 2023). Alterations in the expression or function of these proteases under pathological conditions are intricately linked to the onset and progression of cardiac diseases including atrial fibrillation (AF), ventricular hypertrophy, HF, cardiomyopathy, hypertensive heart disease, MI, and coronary atherosclerotic heart disease (Rizza et al., 2015; Selejan et al., 2018; Yang and Khalil, 2022; Ji et al., 2024; Ji et al., 2025; Li et al., 2025). For example, upregulation of ADAM17 miRNA, which is associated with increased cardiac fibrosis and cardiomyocyte (CM) apoptosis, has been documented in the hearts of mice with diabetic cardiomyopathy (Xue et al., 2022). In advanced-stage dilated cardiomyopathy, *ADAM15* deficiency exacerbates decompensated myocardial hypertrophy (Aujla et al., 2023). Furthermore, ADAMs are crucial in cardiovascular diseases and modulate processes, such as inflammation, angiogenesis, metabolism, cell proliferation, migration, and ion channel function (Xiao et al., 2014; Kawai et al., 2021).

As a high-energy organ, the heart requires substantial amounts of adenosine triphosphate (ATP) to sustain its contractile function, which relies mainly on mitochondrial oxidative phosphorylation and glycolysis (Lopaschuk et al., 2021). The mitochondrion is a double-membrane organelle ubiquitous in eukaryotic cells. It primarily comprises the inner and outer membranes, intermembrane space, and matrix (El-Hattab et al., 2017). Oxygen, glucose, fatty acids, and other nutrients essential for cardiac activity are utilized in oxidative phosphorylation, which generates ATP and contributes to the formation of reactive oxygen species (ROS). Although ROS is crucial for signal transduction, excessive ROS production triggered by certain stimuli can lead to mitochondrial and cytoplasmic oxidative stress (OS). This stress can disrupt normal ion channel functions and other vital cellular activities, potentially resulting in heart disease (Wang et al., 2023). The mitochondrial quality control system encompasses mitochondrial dynamics, mitophagy, and mitochondrial biosynthesis, all of which are integral to maintaining normal mitochondrial morphology. Beyond energy production, mitochondrial quality control is intricately linked to biological processes, such as OS, ferroptosis, apoptosis, and metabolic reprogramming in the heart (Disatnik et al., 2013; Nizami et al., 2022; Yan et al., 2024). Mitochondria are semi-autonomous organelles that are essential for numerous biological functions that rely on the integrity of mitochondrial DNA within the mitochondrial matrix (Wang et al., 2021). Studies have demonstrated that mutations in mitochondrial DNA can precipitate HF and coronary atherosclerotic heart disease (Corral-Debrinski et al., 1992; Zhang et al., 2024).

Although increasing evidence indicates that ADAM family proteases and their substrates are involved in the regulation of cardiac ion channels, relevant studies are limited. Most studies have focused on the regulation of ADAM family members or their specific substrates in a single ion channel. However, systematic reviews and summaries of how ADAM-mediated proteolytic events synergistically regulate different ion channel families and participate in cardiac electrophysiological remodeling are lacking. Therefore, the existing evidence should be integrated and discussed to deepen the understanding of the mechanism of ADAMs and their substrates involved in the pathogenesis of heart diseases and to provide a theoretical basis for the development of related therapeutic strategies.

In this review, we systematically summarized the existing evidence on the role of the ADAM family and its substrates in the regulation of cardiac excitatory and non-excitatory ion channels and the potential role of mitochondrial dysfunction in cardiac remodeling. In addition, we summarized the current drug treatments and emerging therapeutic strategies for ion channel dysfunction, to provide a more comprehensive perspective on the understanding of ADAM-mediated cardiac electrophysiological changes and to provide directions for related basic and translational research in the future.

## 2 Overview of ADAMs in the heart

Within the metalloproteinase M12B carbamate subfamily, 22 ADAM proteins have been identified in humans, namely, ADAM1, 2, 3B, 7, 8, 9, 10, 11, 12, 15, 17, 18, 19, 20, 21, 22, 23, 28, 29, 30, 32, and 33. Notably, only 13 ADAMs possess hydrolase activity (Bode et al., 1993; Edwards et al., 2008; Kawai et al., 2021). In cardiac tissues, a diverse array of cell types express ADAM proteins, including ADAM8, 9, 10, 12, 15, 17, 19, and 33 (Kawai et al., 2021). Notably, ADAM9, 10, 12, and 17 have been detected in various CMs, as well as in immune and non-immune cells, such as endothelial cells (ECs), fibroblasts, neutrophils, macrophages, and monocytes (Zhang et al., 2010; Nakamura et al., 2020; Adu-Amankwaah et al., 2021a; Kawai et al., 2021). Owing to their proteolytic and adhesive characteristics, cardiac ADAMs are crucial in modulating signaling pathways and ensuring proper cardiac development through interactions with cell surface-associated receptors. Nevertheless, in pathological states, the deficiency or overexpression of ADAMs can adversely affect cardiac health (Adu-Amankwaah et al., 2021a; Adu-Amankwaah et al., 2021b). ADAM10 and ADAM17, in particular, exhibit closely related structural and functional attributes (Matthews et al., 2017), leading to a shared repertoire of substrates, including cluster of differentiation 44, heparin-binding epidermal growth factor (EGF)-like growth factor (HB-EGF), C-X3-C motif chemokine ligand 1, interleukin (IL)-6 receptor, tumor necrosis factor-alpha (TNF-α), Klotho, and receptor activator of nuclear factor kappa-Β ligand, all of which are integral to cardiac development (Kawai et al., 2021). The structural integrity of cardiac cells is preserved through the Ca^2+^-dependent homophilic cell-cell adhesion mediated by cadherins, which serve as the primary substrates for ADAM9, 10, and 15 (Craig et al., 2010; Kawai et al., 2021). ADAM19, also known as Meltrin β, plays a crucial role in the development of the endocardial cushion and acts *via* substrates such as neuregulin-1 and receptor activator of nuclear factor kappa-Β ligand (Chesneau et al., 2003; Kurohara et al., 2004; Lange and Yutzey, 2006; Combs and Yutzey, 2009; Kawai et al., 2021). Kurohara et al. demonstrated that mice lacking *Meltrin β* exhibit ventricular septal defects and valvular hypoplasia, with most of the affected mice dying shortly after birth (Kurohara et al., 2004). Under pathological cardiac conditions, ADAM12 participates in disease progression through its proteolytic activity. For example, in a mouse model of cardiac hypertrophy, ADAM12 cleaves the precursor of HB-EGF, causing an increase in soluble HB-EGF. This further activates the EGF receptor, thereby initiating cellular responses such as increased protein synthesis and cell enlargement, ultimately resulting in cardiac hypertrophy (Asakura et al., 2002). Moreover, the knockout of *ADAM9* in mice infected with the encephalomyocarditis virus exacerbates myocardial interstitial edema, thereby contributing to the development of dilated cardiomyopathy (Bazzone et al., 2024).Studies have shown that, under normal conditions, ADAM9−/− mice, ADAM15−/− mice and ADAM8−/− mice are all viable and exhibit no obvious cardiac phenotypic defects (Weskamp et al., 2002; Horiuchi et al., 2003; Kelly et al., 2005). In contrast, deletion of *ADAM10* and *ADAM17* in ECs leads to cardiac abnormalities, such as impaired remodeling of the semilunar valves and cardiac dysfunction, in adult mice (Wilson et al., 2013; Farber et al., 2019). Additionally, increasing evidence suggests that cardiac ADAMs and their substrates are crucial for various essential physiological functions of the heart, including the regulation of cardiac ion channels through the modification of specific genes encoding these channels. The roles of ADAM-related substrates in cardiac function are shown in Table 1.

**TABLE 1:** The roles of various ADAM substrates in the heart.

ADAM protein type	Substrate	Effect	References
ADAM10, ADAM17	Cluster of differentiation44	Promotes the development and elongation of ventricular valve leaflets and maintains the integrity of semilunar valve leaflets.	Nagano et al. (2004), Monaghan et al. (2016)
	HB-EGF	Promotes vascular smooth muscle cell migration; increased expression induces cardiac hypertrophy.	Bakken et al. (2009), Zeng et al. (2013),
	C-X3-C motif chemokine ligand 1	Promotes neutrophil infiltration and migration within CMs, thereby exacerbating the inflammatory response.	Klapproth et al. (2022)
	TNF-α	ADAM17 promotes the conversion of transmembrane TNF-α to the secreted form. Secreted TNF-α leads to ventricular dilation.	Dibbs et al. (2003)
	Klotho	Suppresses the expression of intercellular adhesion molecule-1 and vascular cell adhesion molecule-1 in mouse thoracic aortic ECs, thereby alleviating the development of atherosclerosis.	Maekawa et al. (2009), Saar-Kovrov et al. (2020)
	Receptor activator of nuclear factor kappa-Β ligand	Induce myocardial cell death, recruit and activate leukocytes, thereby directly and indirectly causing cardiac injury.	Singh et al. (2018), Wang et al. (2020b)
ADAM10	Cadherins	Expression is increased in dilated cardiomyopathy.	Li et al. (2017)
ADAM9	Cadherins	*Adam9* gene knockout mice survive.	Weskamp et al. (2002)
ADAM15	Cadherins	*Adam15* gene knockout mice survive.	Ham et al. (2002), Horiuchi et al. (2003)
ADAM19	Neuregulin-1	Exerts a protective effect during myocardial stress; participates in cardiac development.	Kurohara et al. (2004), Tsai et al. (2025)
ADAM12	HB-EGF	Promotes epidermal growth factor receptor transactivation and induces cardiac hypertrophy.	Asakura et al. (2002)

## 3 Regulatory mechanisms of ADAMs and their substrates in cardiac VGCCs

**FIGURE 1:**
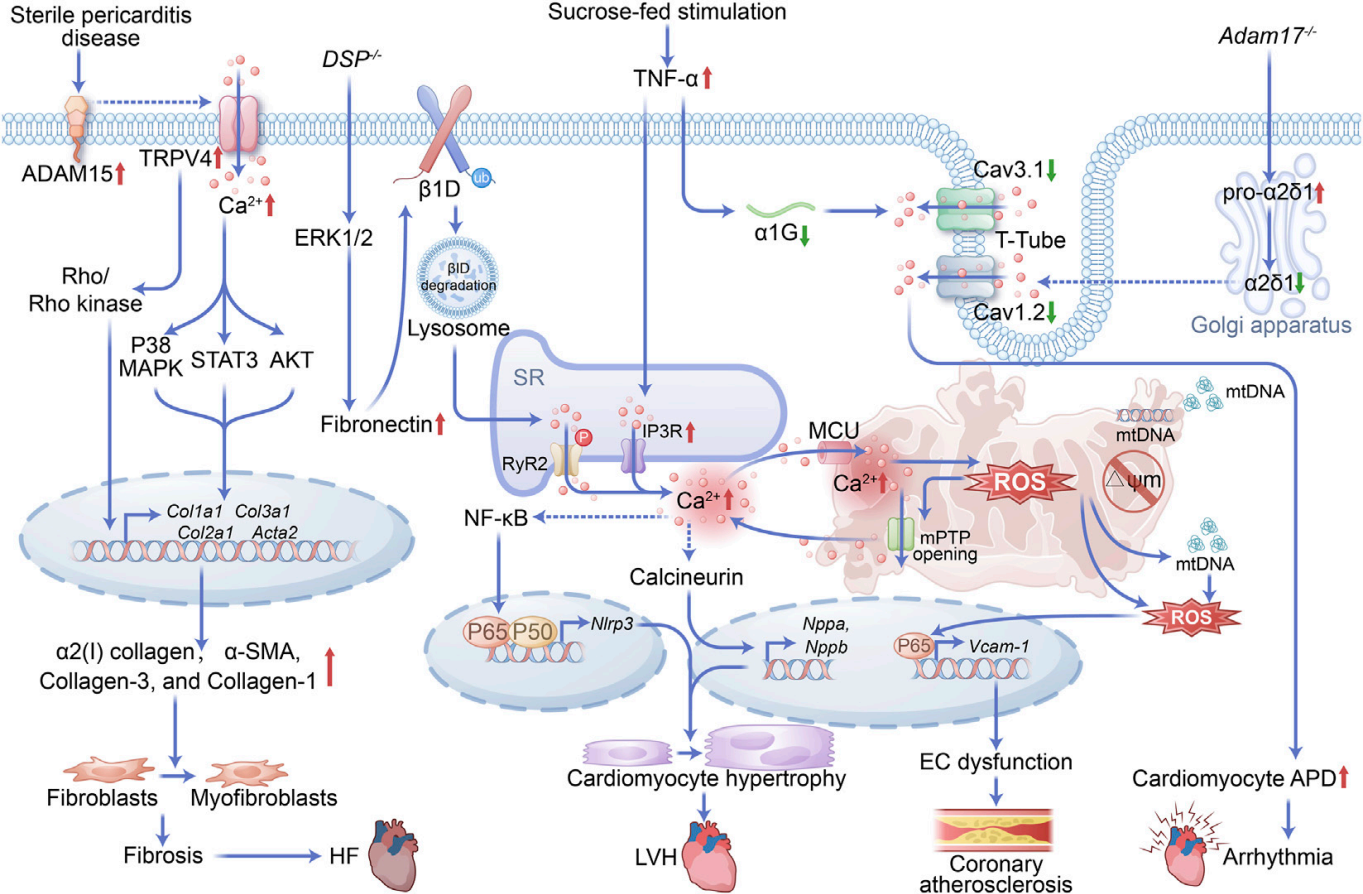
Regulation of cardiac Ca^2+^ channels by ADAMs and their substrates. Sterile pericarditis indirectly activates TRPV4-mediated Ca^2+^ influx by upregulating the expression of ADAM15. This promotes Col I/III and α-SMA expression by activating the Rho/Rho kinase, p38 MAPK, STAT3, and AKT3 signaling pathway. This process induces fibrosis in fibroblasts and ultimately leads to HF. Knockout of *DSP* activates the expression of fibronectin through the phosphorylation of ERK1/2, resulting in the degradation of lysosomal β1D. Reduced β1D leads to the phosphorylation of downstream RyR2. Similarly, under sucrose stimulation, the level of TNF-α increases, and the IP3R on the SR is activated, exacerbating cytoplasmic Ca^2+^ overload. This further indirectly activates the NF-κB signaling pathway and promotes calcineurin activation, leading to CM hypertrophy and the occurrence of LVH. TNF-α also decreases α1G expression, causing downregulation of Cav3.1, and knockout of *ADAM17* hinders α2δ-1 generation in the Golgi, indirectly leading to the downregulation of Cav2.1. During these processes, elevated intracellular Ca^2+^ enters mitochondria through the MCU, causing mitochondrial Ca^2+^ overload and resulting in increased mitochondrial ROS production, accompanied by mPTP opening. Changes in the mitochondrial structure cause significant mtDNA and ROS leakage, upregulation of p65 expression, and increased VCAM-1 expression, leading to EC dysfunction and coronary atherosclerosis. ADAM15, A Disintegrins and metalloproteinases 15; AF, atrial fibrillation; Ca^2+^, calcium ion; Ca_V_, voltage-gated calcium channels; Col1a1/Col2a1/Col3a1,Collagen type I/II/III alpha 1 chain; DSP, Desmoplakin; ERK1/2, Extracellular signal–regulated kinase 1 and 2; HF, heart failure; IP3R, inositol 1,4,5-triphosphate receptor; MCU, Mitochondrial calcium uniporter; mPTP, mitochondrial permeability transition pores; NF-κB, Nuclear factor kappa-B; NLRP3, NLR Family Pyrin Domain Containing 3; ROS, reactive oxygen species; RyR2, ryanodine receptor 2; α-SMA, alpha-smooth muscle actin; STAT3, Signal transducer and activator of transcription 3; TNF-α, tumor necrosis factor-alpha; TRPV4,Transient receptor potential vanilloid sub type 4.

Excitatory cardiac Ca^2+^ channels, including L- and T-type Ca^2+^ channels (LTCC and TCC, respectively), are a class of multi-subunit transmembrane proteins that participate in many important physiological processes, such as AP formation, excitation-contraction coupling, and gene transcriptional regulation, by mediating Ca^2+^ transport across the membrane (Shah et al., 2022). LTCCs are ubiquitously expressed in all cardiac cell types, whereas TCCs are predominantly found in the sinoatrial nodes and atrial CMs, with little to no expression in the ventricular CMs of healthy adults. The Cav1.2 channel, a cardiac LTCC, is primarily encoded by *CACNA1C*. Its function is dynamically regulated by protein phosphatases and is crucial in excitation-contraction coupling (Yu et al., 2016; Herold et al., 2023). Small amounts of Ca^2+^ entering the intracellular space *via* LTCCs during an AP activate RyR2 receptors in the SR and induce the release of large amounts of Ca^2+^ that ultimately trigger myocardial contractions (Bers, 1991; Herold et al., 2023). LTCC and TCC dysfunctions are closely related to a variety of cardiovascular diseases such as AF, Brugada syndrome (BrS), long QT syndrome (LQTS), angina pectoris, and sudden cardiac death (Shah et al., 2022). The mechanisms by which ADAMs and their substrates regulate Ca^2+^ channels are illustrated in Figure 1.

### 3.1 ADAM upregulation enhances cardiac VGCCs

Previous studies have suggested that ADAM family members and their substrates are involved in the regulation of cardiac Ca^2+^ homeostasis and are closely related to the occurrence and development of myocardial electrical remodeling and arrhythmias. However, evidence of the direct regulation of myocardial VGCCs by ADAMs is scant. The existing mechanisms are mainly derived from heterologous expression in cell lines and animal models, and CM-based verification has been limited.

The α2δ subunit is an important auxiliary subunit of Cav1 and Cav2 channels, and its mature processing is essential for the membrane localization and functional maintenance of Ca^2+^ channels. Kadurin et al. found that a reduction in ADAM17 expression in the HEK cell line leads to proteolysis of the α2δ precursor in the Golgi or cell membrane, reduced transport of functional Ca^2+^ channels to the cell membrane, and ultimately a decrease in the Ca^2+^ current density (Kadurin et al., 2022). Cav1.2 is the main source of extracellular Ca^2+^ influx in CMs during excitation-contraction coupling, and its dysfunction is closely related to a variety of arrhythmia diseases. For instance, after MI in mice, decreased Cav1.2 protein expression in the infarct border zone leads to reduced Ca^2+^ influx, an abnormal action potential duration (APD), and a predisposition to early and delayed afterdepolarizations, thereby causing ischemic arrhythmias (Wang et al., 2025a). Therefore, ADAM17 may also mediate α2δ splicing in CMs, and its abnormal splicing function may participate in cardiac electrical remodeling by affecting Cav1.2 function.

In addition to its effects on Ca^2+^ handling in the sarcolemma and SR, ADAM-mediated Cav1.2 dysfunction may also promote cardiac electrical and structural remodeling by amplifying mitochondrial Ca^2+^ stress, ultimately increasing the risk for arrhythmias (Kadurin et al., 2022; Peana et al., 2022; Wang et al., 2025a). Mitochondria are the primary organelles for cardiac excitation-contraction coupling; excess cytosolic Ca^2+^ is rapidly taken up by mitochondria *via* the mitochondrial Ca^2+^ uniporter, particularly at mitochondrial-SR contact sites (Brookes et al., 2004; De Stefani et al., 2011). Physiological mitochondrial Ca^2+^ uptake supports ATP production, whereas pathological Ca^2+^ overload overwhelms the buffering capacity of mitochondria, leading to mitochondrial Ca^2+^ accumulation and bioenergetic stress (Murphy and Steenbergen, 2008; Gunter and Sheu, 2009). This overload facilitates ROS overproduction, disrupts the function of the mitochondrial electron transport chain, and increases the sensitivity of the mitochondrial permeability transition pores (mPTP) to opening (Rizzuto et al., 2012; Bernardi et al., 2015; Halestrap and Richardson, 2015). Persistent mPTP opening leads to mitochondrial membrane potential depolarization, impaired ATP synthesis, and the release of proapoptotic factors (Rizzuto et al., 2012). Mitochondrial dysfunction significantly exacerbates Ca^2+^ imbalance by promoting Ca^2+^ leakage into the cytoplasm, thereby amplifying cytoplasmic Ca^2+^ overload and reinforcing the self-perpetuating cycle of Ca^2+^ handling disorders (Wehrens et al., 2005). This mitochondria-centered Ca^2+^ amplification loop is particularly detrimental to CMs, as elevated cytoplasmic Ca^2+^ enhances spontaneous SR Ca^2+^ release through the ryanodine receptor type 2 (RyR2), thereby increasing delayed afterdepolarizations and arrhythmogenic triggers. Concurrently, mitochondrial ROS serve as signaling mediators that further modify Ca^2+^ channels and Ca^2+^-processing proteins through redox-dependent mechanisms, exacerbating electrical instability and structural remodeling (Hund and Mohler, 2015; Muszyński and Bonda, 2021). Excessive release of ROS due to mitochondrial Ca^2+^ overload also contributes to the pathogenesis and progression of heart disease by altering the biophysical and biochemical properties of Na^+^ channels. In neonatal mouse ventricular CMs, exposure to ROS results in the inhibition of Nav1.5, through oxidation of the cysteine residue C373 located in the Nav1.5 pore region and increase susceptibility to arrhythmia (Schneider et al., 2024). This promotes the reverse mode of the Na^+^/Ca^2+^ exchanger (NCX) channel protein on the cell membrane, leading to an increase in intracellular Ca^2+^ concentration and forming a vicious cycle. (Kohlhaas and Maack, 2010). Other studies have also shown that in apo-KO mice treated with transverse aortic constriction, mitochondrial Ca^2+^ concentration was increased, ROS production was increased and leakage into the cytoplasm, which induced the activation of p38 MAPK and P65 signaling pathways in the cytoplasm, promoted the transcription of Vcam-1, and upregulated the adhesion molecule VCAM-1 on the cell membrane. Induction of macrophage aggregation and arterial plaque formation (Zhang et al., 2023b). In summary, ADAM-driven alterations in Ca^2+^ channel activity not only disrupt SR Ca^2+^ influx but also propagate mitochondrial Ca^2+^ stress, forming a pathological feedforward loop that accelerates CM dysfunction, fibrosis, and susceptibility to arrhythmias (Schneider et al., 2024).

### 3.2 Role of ADAM-related substrates in VGCCs in the heart

In addition to the regulation of cardiac ion channels by ADAM, many studies have focused on the regulation of Ca^2+^ channels by ADAM substrates. In this section, we mainly focused on the effects of TNF-α and integrins on the function and structure of VGCCs and the subsequent effects on the excitation-contraction coupling process in the myocardium.

#### 3.2.1 TNF-α upregulation inhibits cardiac VGCCs

TNF-α is an important substrate of ADAM17 and has been confirmed to be involved in the occurrence and development of various cardiovascular diseases such as AF, myocardial hypertrophy and HF. Clinical studies have shown that the mRNA and protein expression levels of TNF-α are typically elevated in the right atrial tissue of patients with AF. In addition, *in vitro* experiments with HL-1 cells have demonstrated that a concentration-dependent increase in TNF-α reduces the mRNA expression of the TCC α1G subunit and inhibits the voltage-dependent inactivation of TCCs, ultimately leading to a decrease in the TCC current. Such alterations in Ca^2+^ currents can disrupt the normal excitability, conductivity, and repolarization of atrial CMs, thereby facilitating electrical remodeling and directly contributing to AF initiation and maintenance (Rao et al., 2016). Since Ca^2+^ influx mediated by VGCCs ultimately translates into cellular effects *via* SR Ca^2+^ release, the regulation of RyR-mediated Ca^2+^ release by TNF-α may also amplify its effects on cardiac Ca^2+^ homeostasis and electrical remodeling. In neonatal rat ventricular CMs cultured with TNF-α, the expression of the inositol 1,4,5-trisphosphate receptor in the endoplasmic reticulum (ER) and of RyR in the SR facilitates Ca^2+^ release from the SR, contributing to cardiac hypertrophy (Wang et al., 2017). Aberrant RyR2 function in ventricular CMs of mice with transverse aortic constrictions leads to pathological Ca^2+^ release, which activates calcineurin. This upregulates both the pro-hypertrophic calcineurin/nuclear factor of activated T cells 3 pathway and the proinflammatory TNF-α/nuclear factor kappa-B/NOD-, LRR-, and pyrin domain-containing protein 3 pathway, culminating in cardiac hypertrophy (Gao et al., 2024).

#### 3.2.2 Integrin downregulation inhibits Ca^2+^ signal transduction associated with VGCCs

During the coupling of cardiac excitation and contraction, a small amount of Ca^2+^ influx mediated by LTCCs activates RyR2 receptors in the SR, triggering Ca^2+^ release that amplifies the intracellular Ca^2+^ signal and triggers myocardial contractions. Therefore, although RyR2 is not a VGCC, RyR2-mediated SR Ca^2+^ release is an important component of Ca^2+^ signal transduction downstream of LTCCs, and RyR2 dysfunction can further amplify the pathological Ca^2+^ signal mediated by VGCCs. Integrins are important adhesion molecules connecting the extracellular matrix and cytoskeleton that not only participate in mechanical signal transduction but also play important roles in the maintenance of Ca^2+^ homeostasis in CMs (Wang et al., 2025b). Integrins are substrates of ADAM17 (Fedak et al., 2006; Kawai et al., 2021). RGD-containing peptides trigger downstream signaling pathways by binding to integrin receptors on the CM surface. While this process enhances the uptake and release efficiency of Ca^2+^ in the SR, it also promotes the co-localization of LTCCs with RyR2 to achieve more efficient excitation-contraction coupling (Wang et al., 2022) However, significant downregulation of integrin β1D is a key pathological feature of arrhythmogenic right ventricular cardiomyopathy (ARVC) (Wang et al., 2020a). An research have shown that desmosome mutations promote B1D degradation by the ubiquitin-lysosomal pathway through upregulation of pERK1/2 and fibronectin in left ventricular tissue from patients with ARVC; furthermore, in ventricular muscle from adult B1D-specific knockout mice, deletion of B1D directly leads to RyR2 Ser2030 hyperphosphorylation in response to b-adrenergic stimulation, resulting in SR Ca^2+^ leakage, delayed posterior depolarization, and ultimately catecholaminergic polymorphic ventricular tachycardia in ARVC (Wang et al., 2020a).

## 4 Regulatory mechanisms of ADAMs and their substrates in cardiac excitable K^+^ channels

**FIGURE 2:**
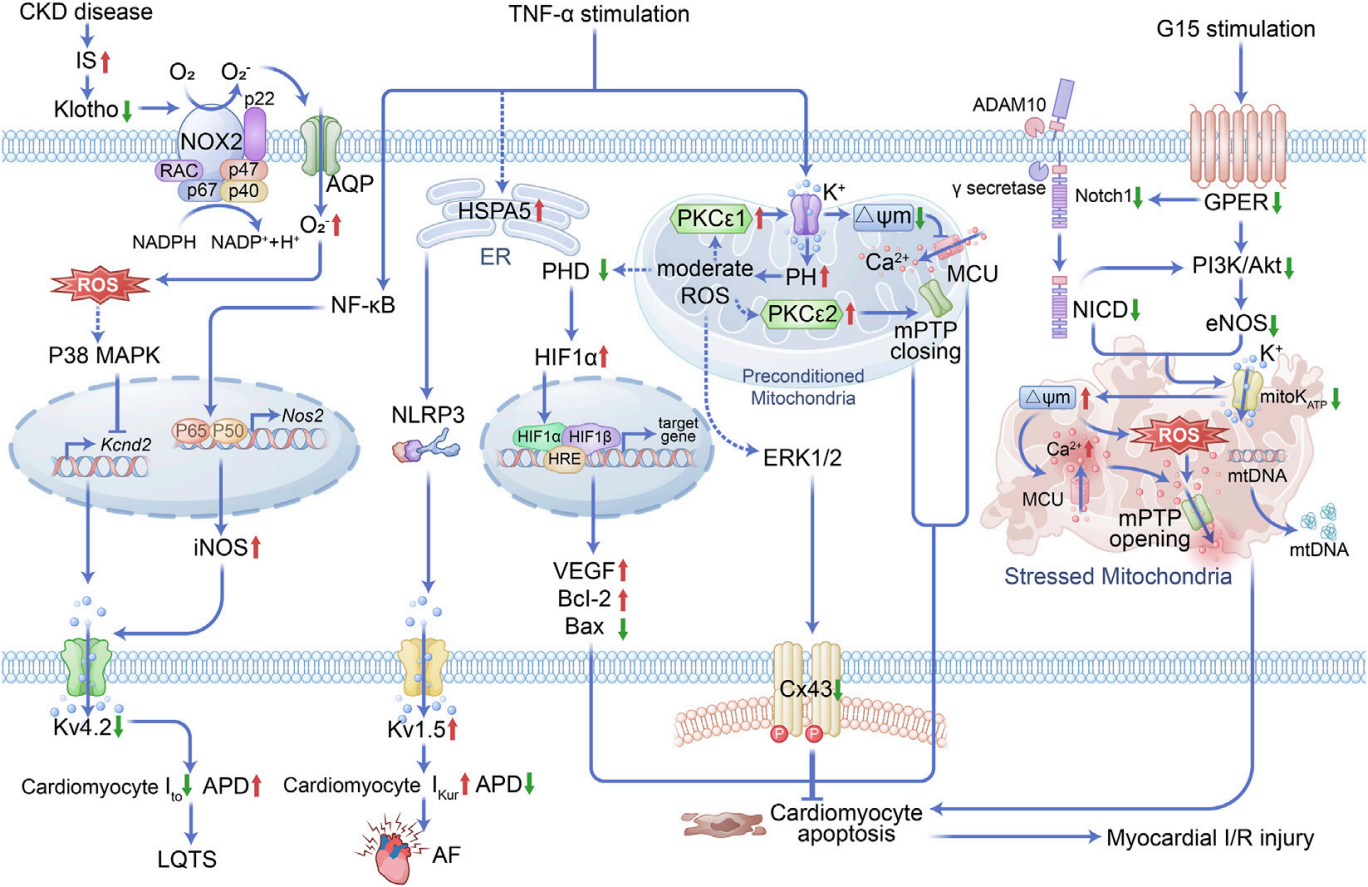
Regulation of cardiac K^+^ channels by ADAMs and their substrates. In CKD, increased IS levels inhibit Klotho expression and activate NOX2, which induce oxidative stress and indirectly activate p38 MAPK. Activated p38 MAPK reduces Kv4.2 expression by inhibiting the transcription of *KCND2*. Concurrently, TNF-α stimulation activates NF-kB and upregulates the expression of iNOS, which similarly reduces Kv4.2 levels. This leads to decreased *I*_*to*_, prolonged the APD, and an increased susceptibility to LQTS. Furthermore, TNF-α stimulation indirectly induces ER stress, which activates the NLRP3 inflammasome and upregulates Kv1.5 expression. This results in an increase in *I*_*Kur*_ and shortening of APD, thereby promoting the development of AF. During ischemic preconditioning, the opening of mitoK_ATP_ induced by TNF-α not only reduces MMP, thereby decreasing Ca^2+^ influx into the mitochondrial matrix via the MCU, but also leads to alkalization of the mitochondrial matrix. This alkaline environment reduces the local proton availability, thereby causing a highly reduced state of respiratory chain complex I, leading to the moderate production of ROS. On the one hand, this indirectly activates PKCε1, which perpetuates mitoK_ATP_ opening through a positive feedback loop and also prevents I/R injury by indirectly inhibiting PHD activity to activate the HIF-1/HRE pathway and by indirectly suppressing PKCε2-mediated opening of the mPTP. Additionally, appropriate levels of mitochondrial ROS indirectly activate the MEK/ERK pathway and promote the phosphorylation of Cx43. This reduces the permeability of GJIC, thereby limiting the spread of I/R injury signals. Conversely, G15 stimulation exacerbates I/R injury in cardiomyocytes by blocking the Notch1/PI3K/Akt/eNOS/mitoK_ATP_ signaling axis, leading to excessive ROS production and subsequent mitochondrial damage. AF, atrial fibrillation; APD, action potential duration; CKD, chronic kidney disease; Cx43, Connexin 43; ER, endoplasmic reticulum; GJIC, gap junctional intercellular communication; HIF-1/HRE, hypoxia-inducible factor 1/hypoxic response element; *I*_*Kur*_, ultra-rapid delayed rectifier K^+^ current; iNOS, inducible nitric oxide synthase; I/R, ischemia-reperfusion; IS, indoxyl sulfate; *I*_*to*_, transient outward K^+^ current; LQTS, long QT syndrome; MCU, Mitochondrial calcium uniporter; MEK/ERK, mitogen-activated protein kinase kinase/extracellular signal-regulated kinase; mitoK_ATP_, mitochondrial ATP-sensitive K^+^ channels; MMP, mitochondrial membrane potential; mPTP, mitochondrial permeability transition pores; NF-kB, Nuclear factor kappa-B; NLRP3, NOD-, LRR- and pyrin domain-containing protein 3; NOX2, NADPH oxidase 2; p38 MAPK, p38 mitogen-activated protein kinase; PHD, prolyl hydroxylase; PI3K/Akt, Phosphatidylinositol 3-Kinase/Protein Kinase B Signaling Pathway; PKCε1, protein kinase C epsilon 1; PKCε2, protein kinase C epsilon 2; ROS, reactive oxygen species; TNF-α, tumor necrosis factor-alpha.

Excitable K^+^ channels in the heart maintain normal electrical activity and systolic function by regulating the resting potentials and the repolarization of APs in CMs and include voltage-gated potassium channels (Kv), such as Kv1.x, Kv2.x, Kv4.x, Kv7.x, and Kv11.x, and inwardly rectified potassium channels (Kir), such as Kir2.x, Kir3.x, and Kir6.x (Schmitt et al., 2014). ADAMs and their substrates significantly influence the cardiac electrophysiological activity by modulating specific Kv, including Kv1.1, Kv4.2, and Kv1.5. Notably, the absence of the Kv1.1 protein, encoded by *KCNA1*, can lead to cardiac autonomic nervous regulation abnormalities, resulting in neurogenic cardiac dysfunction. (Glasscock et al., 2010). Furthermore, aberrant expression of Kv4.2 and Kv1.5 is closely associated with the onset and progression of arrhythmogenic conditions such as AF and acquired LQTS (Fernández-Velasco et al., 2007; Scott et al., 2021). The mechanisms by which ADAMs and their substrates regulate K^+^ channels are illustrated in Figure 2.

### 4.1 ADAM downregulation inhibits cardiac excitable K^+^ channels

Although the direct mechanism underlying the interaction between ADAM and K^+^ channels in CMs remains unknown, the function of these cells is intricately regulated by the autonomic nervous system. Alternatively, ADAMs may indirectly influence the electrophysiological activity of CMs by modulating K^+^ channels in neurons. The ADAM23-LGI3 interaction is crucial for the aggregation of the Kv1.1 complex in the presynaptic region of myelinated axons, helping to regulate the refractory period and ensure the generation of high-frequency APls (Kozar-Gillan et al., 2023). Studies involving *ADAM23* knockout mice demonstrated that the complete absence of ADAM23 in the vagal axon membrane impedes the accumulation of leucine-rich glioma inactivated 1 and 2 in the juxtaparanodal region (Kozar-Gillan et al., 2023). In addition, deletion of Kv1.1 in a mouse model of seizures results in vagal hyperactivity and an increased risk of sudden unexpected death in epilepsy (Glasscock et al., 2010). Therefore, ADAM23 deficiency may cause abnormal parasympathetic innervation by disrupting the formation and homeostasis of Kv1 channels in vagus nerve axons, leading to primary neurogenic cardiac dysfunction.

### 4.2 Role of ADAM-related substrates in cardiac excitable K^+^ channels

ADAM has various substrates in the heart. In terms of cardiac excitable K^+^ channels, we mainly investigated the effects of substrates TNF-α, N-cadherin, and Klotho on K^+^ channels.

#### 4.2.1 TNF-α upregulation regulates cardiac excitable K^+^ channels

For the ADAM substrate TNF-α, few studies have reported a direct mechanism for regulating excitable cardiac K^+^ channels; in adult rat ventricular CMs, exposure to TNF-α upregulates the expression of inducible nitric oxide synthase, thereby significantly enhancing nitric oxide synthesis. The subsequent interaction with the superoxide anion produces peroxynitrite, a potent oxidizing agent, which subsequently results in reduced Kv4.2 expression, decreased transient outward K^+^ current density, prolonged APD, and an increased susceptibility to arrhythmias (Fernández-Velasco et al., 2007). Studies of indirect mechanisms have shown that atrial CMs from adult mice with high-fat diet-induced obesity have significantly elevated protein levels of heat shock protein family A member 5, leading to ER stress and subsequently promoting the assembly of the NOD-, LRR-, and pyrin domain-containing protein 3 inflammasome. The activated inflammasome facilitates the upregulation of Kv1.5, resulting in a shortened effective refractory period in the atria and increased susceptibility to AF (Scott et al., 2021). TNF-α is elevated in obese mice, induces ER stress, and therefore may also participate in this process in an upstream pathway (Petroni et al., 2022). In contrast, Zhou et al. observed an upregulation of TNF-α expression in atrial CMs from patients with chronic AF associated with rheumatic heart disease that was accompanied by a marked reduction in Kv1.5 expression (Zhou et al., 2021). To elucidate the underlying mechanism, H9c2 and HL-1 cell lines were treated with varying concentrations of TNF-α for 24 h. The results demonstrated that TNF-α directly reduces Kv1.5 expression. Concurrently, TNF-α activates the protein kinase C alpha (PKCα) signaling pathway and enhances the phosphorylation of the Kv1.5 channel, thereby inhibiting its function and ultimately leading to a decrease in the ultra-rapid delayed rectifier K^+^ current. This discrepancy may stem from differences in disease models and etiologies, the involvement of distinct signaling pathways, and the early compensatory phase Kv1.5 upregulation that occurred in the obesity model, compared to the latter findings that reflected a late decompensatory state in CMs. PKCα regulates Kv1.5 channels through considerably diverse mechanisms. Notably, in atrial CMs from mice with high-fat diet-induced obesity, OS arising from increased NADPH oxidase 2 (NOX2) expression promotes the upregulation of PKCα and Kv1.5 expression. This upregulation ultimately results in an increase in an ultra-rapid delayed rectifier K^+^ current and heightened susceptibility to AF (McCauley et al., 2020). This discrepancy may stem not only from the heterogeneity of upstream signals that activate PKCα but also from the distinct cellular microenvironments under the two pathological conditions, such as the metabolic load versus mechanical stress, which may recruit different auxiliary proteins, ultimately determining the outcome of PKCα regulation of Kv1.5.

#### 4.2.2 N-cadherin knockdown inhibition cardiac excitable K^+^ channels

N-cadherin plays an integral role in regulating Kv. There is direct evidence to suggest that adult mice with cardiac-specific *CDH2* knockout have CMs with markedly prolonged APDs. This prolongation is attributed to disruption of the actin cytoskeleton caused by N-cadherin deficiency, which leads to reduced cortactin levels. This is accompanied by the downregulation of Kv1.5 and its auxiliary subunit protein K^+^ voltage-gated channel subfamily E regulatory subunit 2, resulting in a significant decrease in the density of the slow-inactivating K^+^ current, ultimately culminating in arrhythmias (Cheng et al., 2011).

#### 4.2.3 Klotho downregulation inhibits cardiac excitable K^+^ channels

In patients with chronic kidney disease (CKD), a prolonged QT interval is an independent risk factor for sudden cardiac death and all-cause mortality, and these risks are exacerbated as renal function declines (Liu et al., 2020). Indoxyl sulfate (IS), a protein-bound uremic toxin, is typically elevated in the serum of patients with CKD due to its limited removal by conventional dialysis (Yang et al., 2022). In a neonatal rat model of CKD, the decreased expression of Klotho caused by increased IS leads to the activation of NOX2 in CMs to produce O_2_^−^, which further activates the p38 MAPK pathway and eventually leads to myocardial hypertrophy and aggravates the progression of left ventricular hypertrophy (Yang et al., 2015). Furthermore, studies have reported that IS also activates the ROS/p38 MAPK pathway in CMs of CKD rats and downregulates the expression of K^+^ channel-related subunits such as Kv4.2, Kv4.3, and KChIP2, thereby prolonging APD and QT intervals and increasing the susceptibility to ventricular arrhythmias (Yang et al., 2022). However, there is no direct evidence that IS reduces the activity of excitatory K^+^ channels in CMs by inhibiting Klotho expression. Considering the above evidence, in the state of CKD, IS may increase the risk of arrhythmias in patients with CKD by inhibiting Klotho expression, activating NOX2-mediated oxidative stress and the p38 MAPK signaling pathway, and ultimately downregulating *KCND2* transcription.

## 5 Regulatory mechanisms of ADAMs and their substrates in cardiac VGSCs

**FIGURE 3:**
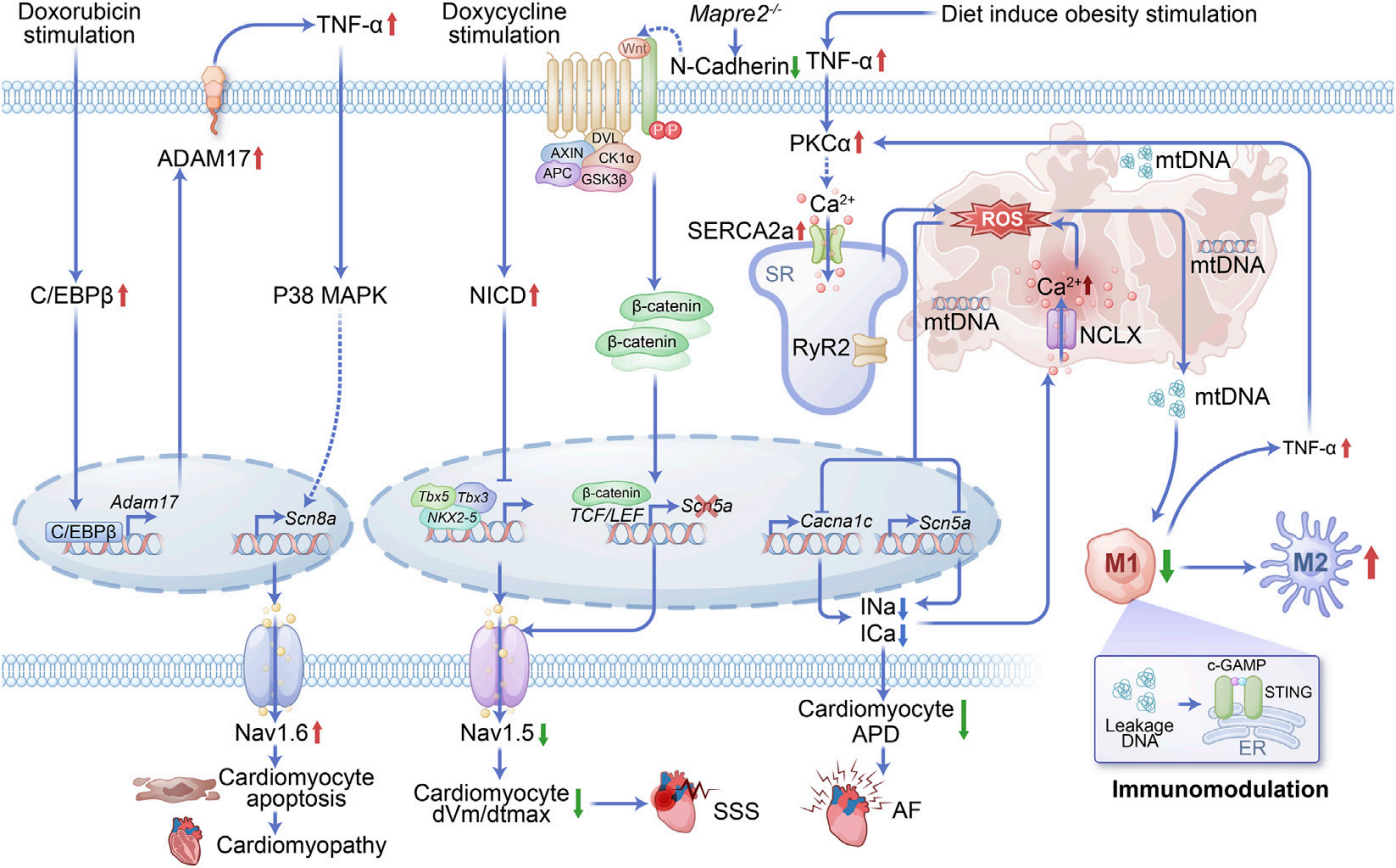
Regulation of cardiac Na^+^ channels by ADAMs and their substrates. Doxorubicin stimulation upregulates C/EBPβ and enhances the transcription of *ADAM17*, which cleaves and releases mature TNF-α. TNF-α then activates p38 MAPK, indirectly promoting the transcription of *SCN8a* and inducing CM apoptosis. Doxycycline stimulation upregulates the expression of the NICD, which subsequently inhibits the transcription of *TBX3, TBX5,* and *NKX2-5*, leading to the downregulation of Nav1.5 expression. Concurrently, the downregulation of N-cadherin expression resulting from *MAPRE2* knockout inhibits *SCN5a* transcription via indirectly activation of the Wnt/β-catenin signaling pathway. The reduced Nav1.5 levels decrease the maximal upstroke velocity of CMs, ultimately leading to SSS. Under conditions of diet-induced obesity, TNF-α activates PKCα, which indirectly induces the activation of SERCA2a on the SR, leading to a significant upregulation of mitochondrial ROS production and the cytoplasmic release of mtDNA. This further inhibits the transcription of *CACNALC* and *SCN5a*, resulting in decreased *I*_*Na*_ and *I*_*Ca*_, a shortened APD, and an increased susceptibility to AF. Simultaneously, the reduced *I*_*Na*_ activates the mitochondrial NCLX, leading to increased mitochondrial Ca^2+^ influx, which further promotes ROS production. The mtDNA released from mitochondria activates the cGAS-STING pathway located on the ER of M1 macrophages. This drives the transformation of M1 macrophages into the M2 phenotype, which in turn produces additional TNF-α, thereby creating a vicious cycle. AF, atrial fibrillation; APD, action potential duration; C/EBPβ, CCAAT/enhancer-binding protein beta; cGAS-STING, cyclic GMP-AMP synthase-stimulator of interferon genes; CMs, cardiomyocytes; ER, endoplasmic reticulum; *I*_*Ca*_, Ca^2+^ current; *I*_*Na*_, fast Na^+^ current; mtDNA, mitochondrial DNA; NCLX, mitochondrial Na^+^/Ca^2+^/Li^+^ exchanger; NICD, Notch intracellular domain; NKX2-5, NK2 homeobox 5; p38 MAPK, p38 mitogen-activated protein kinase; PKCα, protein kinase C alpha; ROS, reactive oxygen species; SERCA2a, sarcoplasmic/endoplasmic reticulum Ca^2+^ATPase 2a; SR, sarcoplasmic reticulum; SSS, sick sinus syndrome; TBX3, T-box transcription factor 3; TBX5, T-box transcription factor 5; TNF-α, tumor necrosis factor-alpha.

In the 1980s, Noda et al. elucidated the topological structure of the α-subunit of VGSCs, thereby laying the groundwork for subsequent research on their functions and regulatory mechanisms (Noda et al., 1984). To date, six Nav subtypes (Nav1.1–Nav1.6) have been identified in human atrial CMs (Maier et al., 2002; Maier et al., 2004; Westenbroek et al., 2013; Nathan et al., 2021). Among these, Nav1.5, encoded by *SCN5A*, is the predominant Nav subtype in mammalian CMs that is primarily localized at the lateral membrane and intercalated discs, where it plays a crucial role in mediating rapid depolarization and facilitating intercellular electrical signal transmission; however, it is entirely absent from transverse tubules (Maier et al., 2004; Kaufmann et al., 2013; Westenbroek et al., 2013). ADAM substrates predominantly influence cardiac electrophysiological activity through the regulation of Nav1.5. Nav1.5 is intricately linked to myocardial excitability and electrical conduction; abnormalities in this channel can lead to primary electrical disorders, such as BrS and progressive cardiac conduction disease, as well as structural heart diseases with established genotype-phenotype correlations, including ARVC(Derangeon et al., 2017; Wilde and Amin, 2018; Rico et al., 2021; Tariq et al., 2024). In contrast, Nav1.6 is primarily regulated directly by ADAMs. The mechanisms by which ADAMs and their substrates regulate Na^+^ channels are illustrated in Figure 3.

### 5.1 ADAM upregulation enhances cardiac VGSCs

Although there is sufficient evidence that ADAMs play an important role in the regulation of cardiac pathophysiology and are involved in the occurrence of AF, myocardial hypertrophy, and acquired LQTS, few studies have focused on the direct regulation of cardiac voltage and Na^+^ channels by ADAMs. Studies have shown that *ADAM17* transcription is enhanced along with the upregulation of nuclear transcription factor C/EBPβ in ventricular myocytes of mice with adriamycin-induced cardiomyopathy. Increased ADAM17 on the cell membrane cleaves tmTNF-α, releases sTNF-α, and further activates the p38 MAPK pathway (Xie et al., 2024). In addition, TNF-α promotes the expression of VGSCs, such as Nav 1.1 and Nav 1.6, by activating the p38 MAPK pathway in mouse cortical neurons (Chen et al., 2015). There is no direct evidence that the ADAM17/TNF-α/p38 MAPK pathway regulates the expression of VGSCs in CMs; however, considering the above evidence, we can speculate that ADAM17 may also affect the expression or function of VGSCs in ventricular myocytes through this signal axis in mice with adriamycin-induced cardiomyopathy and thus contributes to cardiac function deterioration.

### 5.2 Role of ADAM-related substrates in cardiac VGSCs

In this section, we investigated the effects of three ADAM substrates, TNF-α, N-cadherin, and Notch, on cardiac VGSCs.

#### 5.2.1 TNF-α upregulation inhibits cardiac VGSCs

In 3-week-old rabbit ventricular CMs transduced with an adenovirus carrying the *LITAF* gene, overexpression of LITAF was found to inhibit the expression of the ubiquitin ligase neural precursor cell-expressed developmentally downregulated 4 type 2. This inhibition reduces the ubiquitination-mediated degradation of Nav1.5 protein. As a result, these modifications contribute to a reduction in APD and a prolonged QT interval (Turan et al., 2020). Studies have demonstrated elevated PKCα expression in normal rat CMs that are cultured with high concentrations of TNF-α(Jude et al., 2018). Atrial CMs derived from diet-induced obese mice have upregulated PKCα expression and significantly increased SERCA2a expression in the SR. These alterations contribute to enhanced mitochondrial ROS production, elevated NOX2 expression, and mitochondrial OS. Consequently, the protein expression of Cav1.2 and Nav1.5 is reduced, leading to diminished *I*_Ca_ currents and *I*_*Na*_, shortened APD, and the onset of AF (McCauley et al., 2020). Moreover, the reduction in *I*_*Na*_ induced by increased ROS in CMs results in decreased Na^+^ influx into the cytoplasm. This triggers the reverse operation of the mitochondrial Na^+^/Ca^2+^/Li^+^ exchanger, causing mitochondrial accumulation of Ca^2+^, which leads to mitochondrial Ca^2+^ overload and further ROS production, thereby establishing a detrimental feedback loop (Pérez-Hernández et al., 2021). Additionally, mitochondrial OS, driven by elevated mitochondrial ROS in CMs, facilitates mitochondrial DNA damage and release through the upregulation of Bax expression. This process activates the cyclic GMP-AMP synthase-stimulator of interferon genes signaling pathway in macrophages, resulting in the increased production of several inflammatory factors, including TNF-α, IL-1β, and transforming growth factor-alpha. These inflammatory factors further impair Nav1.5 channels in the CM membrane, creating a vicious cycle (Zheng et al., 2024). Based on previous studies, we can speculate that ADAM17 may be involved in the pathophysiological process of atrial electrical and structural remodeling by upregulating the expression of PKCα through its substrate TNF-α in a diet-induced obese mouse model of AF. In addition, OS exacerbated by ADAM17/TNF-α/PKCα axis may act synergistically with mitochondrial Ca^2+^ overload and the inflammatory factor storm to cause the progressive deterioration of cardiac function.

#### 5.2.2 N-cadherin downregulation inhibits cardiac VGSCs

N-cadherin is a crucial adhesion molecule in the intercalated discs of CMs that is essential for maintaining the structural integrity of the myocardium (Chen et al., 2022). Direct experimental evidence demonstrates that induced pluripotent stem cell-derived CMs (iPSC-CMs) from patients with arrhythmogenic right ventricular dysplasia and cardiomyopathy have shown that the R1898H mutation in *SCN5A* leads to a simultaneous reduction in the density of Nav1.5 and N-cadherin protein clusters in the intercalated discs (Te Riele et al., 2017). This observation suggests that N-cadherin plays an important role in the regulation of cardiac VGSCs, thereby contributing to the pathogenesis of cardiac diseases. Ventricular CMs from *MAPRE2* knockout zebrafish and iPSC-CMs with *MAPRE2* knockout have shown a reduction in detyrosinated tubulin levels, which results in N-cadherin mislocalization. This mislocalization subsequently causes Nav1.5 protein dysfunction, decreased *I*_*Na*_ density, impaired ventricular conduction, and the manifestation of a BrS phenotype (Chiang et al., 2024). During the early stages of cardiac development, N-cadherin expression is low in cardiac precursor cells. β-catenin can be anchored to the cell membrane through adhesion junctions, while the appropriate cytosolic levels of free β-catenin can enter the nucleus, activate Wnt signaling to maintain the undifferentiated state of cardiac precursor cells in the precordial region, and promote their proliferation (Soh et al., 2014). However, N-cadherin is highly expressed in mature CMs and tightly anchors β-catenin to the cell membrane, leading to decreased cytosolic free β-catenin and significantly decreased Wnt signaling (Soh et al., 2014; Fan et al., 2018). In iPSC-CMs derived from patients with BrS, the loss of Nav1.5 function caused by *SCN5A* mutations causes nuclear redistribution of β-catenin through interactions between Nav1.5 and β-catenin and leads to abnormal activation of the Wnt/β-catenin pathway. This further represses *SCN5A* transcription, thereby forming a vicious cycle (Cai et al., 2023). Therefore, the loss of N-cadherin in CMs may also promote the abnormal activation of the Wnt/β-catenin pathway by releasing membrane-bound β-catenin and inhibit the transcription of *SCN5A* in CMs, which eventually leads to the occurrence of BrS.

#### 5.2.3 Notch upregulation regulates cardiac VGSCs

There is direct experimental evidence demonstrating that ventricular CMs from an adult transgenic mouse model with inducible, CM-specific overexpression of the Notch1 intracellular domain demonstrate a reduced density of *I*_*Na*_, leading to a decreased rate and amplitude of the AP upstroke (Borghetti et al., 2018). In the atrial CMs of adult mice, doxycycline-induced transient activation of the Notch intracellular domain inhibits the expression of key transcriptional regulators such as *TBX3*, *TBX5*, and *NKX2-5*, which are crucial for sinoatrial node pacemaker function and atrial CMs conduction, respectively. This suppression downregulates *SCN5a* expression, resulting in atrial electrical remodeling characterized by a decreased maximal upstroke velocity of the atrial AP, reduced heart rate, and induction of sinus arrest (Qiao et al., 2017). Zhang et al. demonstrated that in an adult mouse model of MI-induced HF, the mechanism of cardiac radiotherapy involves the activation of the Notch signaling pathway, which upregulates Nav1.5 expression, enhances electrical conduction, and reduces heterogeneity and reentry, thereby inhibiting ventricular tachycardia and delaying HF progression (Zhang et al., 2021). The differential outcomes of these mechanisms may be attributed to the activation of the Notch signaling pathway, leading to distinct outcomes in different cellular models.

## 6 Regulation of non-excitable ion channels by ADAMs and their substrates

Cardiac non-excitable ion channels and their related regulatory components mainly include transient receptor potential (TRP) channels, store-dependent Ca^2+^ entry (SOCE) -related proteins, such as STIM/ORAI, and ATP-sensitive potassium channels (K_ATP_). They play a key role in pathophysiological processes, such as regulating Ca^2+^ signaling, maintaining EC function, regulating vascular tone, and participating in cardiac remodeling (Collins et al., 2013; Earley and Brayden, 2015; Foster and Coetzee, 2016; Bon et al., 2022).

The TRP channels family is a group of Ca^2+^ permeable membrane ion channel proteins, including transient receptor potential vanilloid 4 (TRPV4) and transient receptor potential cation 6 (TRPC6), which are critical in regulating cardiac endothelial Ca^2+^ influx. TRPV4 channels play important roles in the development of AF and other arrhythmic diseases (Mendoza et al., 2010; Li et al., 2021; Chaigne et al., 2023). Atrial myocytes and fibroblasts of adult rats with sterile pericarditis-induced AF show increased expression of the TRPV4 channel, accompanied by increased Ca^2+^ influx and prolonged APD. Simultaneously, increased Ca^2+^ in atrial fibroblasts leads to the activation of p38, AKT, and STAT3. The subsequent increased expression of collagen I, collagen III, α-SMA, and TGF-β leads to atrial fibrosis and the development of AF (Liao et al., 2020). In the fibroblasts of adult mice with MI, TRPV4 upregulates the expression of pro-fibrotic genes *Acta2* and *Col1a2* by activating the Rho/Rho-kinase pathway, promoting the differentiation of cardiac fibroblasts into myofibroblasts, leading to the occurrence of fibrosis and cardiac remodeling, and thereby increasing the susceptibility to HF(Adapala et al., 2020).In the aged mouse model of cardiac ischemia-reperfusion (I/R), hypoosmotic stress during reperfusion can activate TRPV4 channel, leading to a significant increase in Ca^2+^ influx in left ventricular CMs, which induces a large amount of ROS production, further promotes the opening of mPTP and depolarization of mitochondrial membrane potential, and eventually leads to ventricular myocyte apoptosisand increases the risk of arrhythmia after reperfusion (Wu et al., 2017; Peana et al., 2022). Although there is as yet no direct evidence in CMs to suggest that ADAMs are involved in the aforementioned process by regulating TRPV4 channels, studies have shown that, in synovial fibroblasts from patients with rheumatoid arthritis, increased mechanical stress elevates ADAM15 expression levels, thereby promoting an increase in the density of TRPV4 channels on the cell membrane surface, which leads to enhanced Ca^2+^ currents. (Janczi et al., 2021). Therefore, ADAM may also participate in Ca^2+^ homeostasis and cardiac structural remodeling by affecting the function and expression level of TRPV4 in CMs.

K_ATP_ are regulated by the ATP/ADP ratio in cells, and their activity directly reflects the state of cellular energy metabolism (Gross and Fryer, 1999; Foster and Coetzee, 2016; Tinker et al., 2018). K_ATP_ can be divided into two major isoforms according to the sarcolemmal ATP-sensitive K^+^ (sarcK_ATP_) and mitochondrial K_ATP_ (mitoK_ATP_) (Gross and Fryer, 1999; Foster and Coetzee, 2016). ADAMs depend on the substrate TNF-α to regulate mitoK_ATP_ and interact with mitochondrial Ca^2+^ ions to regulate the pathophysiological process of myocardial ischemic preconditioning (IPC). The protective effect of TNF-α during IPC is dependent on the opening of mitoK_ATP_, which leads to mitochondrial matrix alkalinization and ROS production (El-Ani and Zimlichman, 2003; Fu et al., 2003; Fu et al., 2005; Andrukhiv et al., 2006; Matejíková et al., 2009). In rat ventricular myocytes, moderate mitochondrial ROS production inhibits the activity of prolyl hydroxylase and prevents the ubiquitination and degradation of Hif-1α, thus activating the HIF-1/HRE pathway and upregulating the expression of VEGF and Bcl-2. Furthermore, it can enhance the anti-apoptosis ability of CMs (Chen et al., 2021). In addition, ROS in rat ventricular myocytes activate PKCε1 located in the mitochondrial inner membrane, which continuously phosphorylates mitoK_ATP_ in a positive feedback loop and promotes mitoK_ATP_ opening (Costa and Garlid, 2008; Garlid et al., 2013). Simultaneously, PKCε2 is activated, leading to the phosphorylation of the mPTP, thereby inhibiting its opening (Costa and Garlid, 2008; Garlid et al., 2013). In addition, moderate mitochondrial ROS production in ischemic rabbit ventricular myocytes activates the MEK/ERK pathway, leading to the phosphorylation of Cx43 at Ser279/282 and resulting in decreased gap junction intercellular communicationand the reduced spread of damage signals between cells. Thus, the infarct size is reduced (Naitoh et al., 2006). Studies have established that the influx of K^+^ caused by the mitoK_ATP_ opening in IPC reduces the driving force for Ca^2+^ ions to enter the mitochondrial matrix through the mitochondrial Ca^2+^ uniporter (MCU) by reducing the MMP. This significantly reduces the mitochondrial Ca^2+^ overload and maintains normal mitochondrial function during I/R (Wang et al., 2001). Although modest increases in mitochondrial ROS levels during IPC trigger protective pathways, excessive OS caused by the disruption of these pathways has the opposite effect. In spontaneously hypertensive rat ventricular muscles, inhibition of the G protein-coupled estrogen receptor by G15 blocks the cleavage and activation of the ADAM substrate Notch1, reduces the production of Notch intracellular domain, and subsequently, reduces the phosphorylation of the PI3K/Akt and eNOS pathways. Inhibition of this signaling pathway can block mitoK_ATP_ opening, increase the MMP, increase OS, and disrupt mitochondrial function, ultimately leading to CM apoptosis (Rocca et al., 2018).

SOCE is mainly composed of the ER Ca^2+^-sensing stromal interaction molecule 1 (STIM1) and the cell membrane Ca^2+^ channel protein Orai1 (Lewis, 2020). SOCE is overactivated and highly expressed under myocardial I/R conditions. ATP depletion in CMs during ischemia leads to SR Ca^2+^ pump dysfunction and progressive depletion of the SR Ca^2+^ reserve. During reperfusion, the EF-hand domain of STIM1 senses the decrease in Ca^2+^ concentration in the SR lumen caused by the depletion of the SR Ca^2+^ reserve and undergoes a conformational change to interact with Orai1 on the plasma and activate SOCE, leading to increased intracellular Ca^2+^ influx and reperfusion arrhythmia (Collins et al., 2013; Correll et al., 2015). In addition to affecting the intra-mitochondrial Ca^2+^ transport, moderate mitochondrial production of ROS during IPC can also prevent I/R injury by reducing the expression of SOCE. In rat ventricular myocytes, moderate ROS production activates the MAPK/ERK pathway and upregulates STIM1 and Orai1 transcription. Despite the increased expression of STIM1 and Orai1, the effective coupling of STIM1 and Orai1 is reduced due to an imbalance in the STIM1/Orai1 ratio and the abnormal subcellular localization of STIM1, which results in decreased SOCE activity. Sampieri et al. also found that the decrease in mitochondrial Ca^2+^ uptake during IPC leads to the reverse mode of NCX activation, which in turn causes an increase in local Ca^2+^ concentrations and induces Ca^2+^-dependent inactivation of Orai1, ultimately leading to a decrease in SOCE activity (Sampieri et al., 2019). All of the above mechanisms play protective roles in myocardial I/R injury by inhibiting the intracellular Ca^2+^ overload.

## 7 Treatments

With the continuous elucidation of the mechanisms of the regulation of cardiac ion channels by ADAMs and their substrates, researchers have begun to reconsider the impact of some marketed drugs on cardiac electrophysiology. An increasing number of studies have shown that some traditional cardiovascular drugs exert antiarrhythmic effects by regulating the ADAM-related signaling pathway and its substrate-mediated ion channel remodeling, in addition to their classical pharmacological effects. This not only provides a new theoretical basis for understanding the therapeutic mechanism of these drugs but also provides an important direction for ADAM network-based ion channel intervention strategies. Studies of K^+^ channels have shown that Shensongyangxin capsule improves myocardial electrophysiology by protecting vascular endothelial function, inhibiting the release of inflammatory factors such as TNF-α and IL-1β, and inhibiting M1 polarization of macrophages in leptin deficient mice with type 2 diabetes mellitus, thereby upregulating the mRNA expression of Kv4.2 and Kv4.3 channel proteins, improving myocardial electrophysiology, and reducing arrhythmia susceptibility in diabetic mice (Zhang et al., 2023a). The ADAM17 substrate, interferon-gamma (IFN-γ), is predominantly secreted by Th1 cells (Kanzaki et al., 2016). In a rat model of myocardial infarction, valsartan inhibits the secretion of IFN-γ by Th1 cells, thereby lifting the suppression of Kir2.1 protein expression exerted by this cytokine, which in turn restores the IK1 current density in ventricular myocytes within the damaged area and effectively reduces the risk of post-infarction ventricular arrhythmias. (Li et al., 2016). Furthermore, in the context of Na^+^ channel regulation within a MI mouse model, the cardiac conduction velocity can be significantly enhanced through a single 25 Gy dose of cardiac radiotherapy administered 2 weeks post-infarction. This treatment consistently activates the Notch signaling pathway and upregulates the expression of *SCN5a*, indicating a potential therapeutic strategy for the clinical management of refractory scar-related ventricular tachycardia (Zhang et al., 2021). In addition, at the Ca^2+^ channel level, Klotho specifically binds to the third extracellular loop of the TRPC1 channel through its C-terminal region to regulate Ca^2+^ influx and prevent Ca^2+^ overload-induced cardiac over-remodeling (Fakhar et al., 2022). Fakhar et al. suggested that diltiazem and felodipine may play a role similar to that of Klotho in maintaining Ca^2+^ homeostasis (Fakhar et al., 2022).The application of existing therapeutic strategies targeting ADAM and its substrates in diseases associated with abnormal cardiac channel expression are summarized in Table 2.

**TABLE 2:** The existing therapeutic strategy targeting ADAM and its substrates in the application of diseases related to abnormal expression of cardiac channels.

Channel	Strategy	Target	Model disease	Mechanism	Effect	References
Na^+^ channel	Cardiac radiotherapy	Notch	MI mouse model	Promote Notch activation, upregulate *Scn5a* gene expression	Improve post-MI refractory scar-related ventricular tachycardia	Zhang et al. (2021)
K^+^ channel	Shensongyangxin capsule Valsartan	TNF-α IFN-γ	Leptin-deficient mouse model with type 2 diabetes MI rat model	Inhibit the release of TNF-α and IL-1β, and upregulate the mRNA expression of Kv4.2 and Kv4.3 channel proteins. Antagonize the inhibitory effect of IFN-γ on Kir2.1 protein	Reduce susceptibility to arrhythmias Improve arrhythmia after MI	Qiu et al. (2016); Li et al. (2016)

In addition to the in-depth exploration of the mechanisms of existing drugs, the deeper understanding of cardiac ion channel remodeling and the upstream regulatory network have enabled a series of recombinant proteins, bioactive molecules, and specific signal pathway modulators to enter the preclinical stage. These emerging therapeutic strategies can more precisely regulate the expression of ion channels, Ca^2+^ homeostasis, and myocardial electrical activity and show good translational prospects for the treatment of diseases such as arrhythmias and cardiac remodeling. At the level of Ca^2+^ channel regulation, an adult mouse model of MI has demonstrated that treatment with recombinant Klotho stabilizes Ca^2+^ release by inhibiting CaMKII and reducing the abnormal phosphorylation of RyR2, thereby decreasing the risk of arrhythmia onset post-MI (Vázquez-Sánchez et al., 2025). Additionally, in a mouse model of isoprenaline-induced myocardial hypertrophy, combined treatment with Na^+^/K^+^ APTase α2 and Klotho upregulates LTCCs on the cell membrane, inhibits NCX, and reduces the pathological Ca^2+^ influx induced by isoprenaline, thereby blocking the pro-hypertrophic calcineurin-nuclear factor of activated T cells pathway and ultimately alleviating myocardial hypertrophy (Song et al., 2019). In relation to K^+^ channel regulation, astragaloside-IV upregulates Klotho expression in an isoprenaline-induced rat model of bradycardia, thereby increasing HCN4 mRNA expression. This upregulation enhances the I_f_ current and improves the sinoatrial node pacing function (Qiu et al., 2016). Preclinical therapeutic agents targeting ADAMs and their substrates in cardiac ion channels are shown in Table 3.

**TABLE 3:** Preclinical strategies targeting ADAM and its substrates in diseases associated with abnormal cardiac channel expression.

Channel	Target	Model disease	Mechanism	Effect	References
Calcium channel	Klotho Klotho	Adult mouse model of MI ISO-induced mouse model of cardiac hypertrophy	Inhibits CaMKII, reduces abnormal RyR2 phosphorylation, stabilizes calcium release Upregulates LTCC channels, inhibits NCX, reduces calcium influx, blocks Calcineurin- NFAT pathway	Improves cardiac arrhythmias Alleviates cardiac hypertrophy	Vázquez-Sánchez et al. (2025); Song et al. (2019)
Potassium channel	Klotho	Isoproterenol-induced rat model of bradycardia	Upregulates klotho, promotes HCN4 mRNA expression, enhances If current density	Improves sinoatrial node pacing function	Qiu et al. (2016)

Although therapeutic strategies targeting ADAMs and their substrates for cardiac ion channel-related diseases have shown therapeutic potential in animal experiments, most research is still in the preclinical stage, and cardiovascular clinical studies directly targeting ADAMs and their substrates are relatively limited. For example, a novel ADAM10 inhibitor, VTH144, has been shown to significantly reduce neutrophil infiltration into the infarct area and IL-1β-driven inflammation in a mouse model of acute MI by inhibiting ADAM10-mediated CX3CL1 shedding (Klapproth et al., 2026). A close association between the inflammatory microenvironment and the transcription of Nav1.5 and its membrane expression levels has been suggested, and ADAM is involved in the regulation of various inflammatory factors, including TNF-α; however, direct evidence that this drug directly affects the function of cardiac ion channels is lacking. Although most relevant research is still in the preclinical stage, an in-depth understanding of ADAMs and their substrate regulatory network and the development of ADAM modulators with high selectivity and low off-target effects are expected to provide new strategies for the precise treatment of arrhythmias.

## 8 Summary and future perspectives

This review systematically summarized the physiological and pathological roles of the ADAM family and its substrates in the heart, with a focus on their maintenance of cardiac electrophysiological homeostasis by regulating the expression, localization, and function of cardiac ion channels. We further explored their potential mechanisms of action in arrhythmia, myocardial hypertrophy, and HF. Under physiological conditions, ADAMs and their substrates are essential for cell adhesion, signal transduction, and tissue development. However, the abnormal expression of genes encoding ADAMs and the aberrant activity of ADAM proteins can disrupt ion channel protein expression, localization, and function, thereby precipitating cardiac pathologies. Based on current evidence regarding the specific mechanisms by which ADAMs and their substrates, such as TNF-α, Klotho, and N-cadherin, directly or indirectly regulate cardiac ion channels, we focused on their roles in the regulation of Ca^2+^, K^+^ and Na^+^ channels. Regarding Ca^2+^ channels, ADAMs and their substrates cooperatively regulate voltage-gated Ca^2+^ channels and Ca^2+^ release from the SR. The dysfunction of these regulatory mechanisms is a critical contributor to disrupted Ca^2+^ homeostasis, myocardial hypertrophy, fibrosis, and other pathological processes, ultimately promoting the development of arrhythmias and hypertrophic cardiomyopathy. In the context of K^+^ channels, ADAMs and their substrates influence the expression and function of various VGKCs and Kir channels, leading to abnormal repolarization and an elevated risk of various arrhythmic diseases, such as LQTS and AF. At the level of Na^+^ channels, ADAM and its substrates mainly affect the expression, localization, and function of VGSCs, such as Nav1.5, which affect the cardiac excitability and conductivity and are closely related to a variety of primary electrical and structural heart diseases. Many studies have linked mitochondrial dysfunction to the pathophysiology of various diseases such as HF and cardiomyopathy, particularly in the context of cardiac ion channel dysfunction. Furthermore, this review elaborated on the specific mechanisms by which mitochondrial dysfunction contributes to ADAM- and substrate-induced cardiac ion channel dysregulation. Intracellular Ca^2+^ overload induced by ADAMs and their substrates is achieved primarily through disrupted mitochondrial dynamics and OS, which in turn leads to alterations in the density and function of various ion channels on the cell membrane, ultimately resulting in cardiac disorders, such as arrhythmias and myocardial hypertrophy. This review also reveals that dysfunction of K^+^ channels often induces pathological responses by affecting Ca^2+^ homeostasis. In particular, abnormalities in mitochondrial K^+^ channels can cause mitochondrial dysfunction and promote Ca^2+^ influx into mitochondria. However, evidence linking mitochondrial regulation to these mechanisms remains limited and warrants further investigation.

Although evidence regarding the regulation of cardiac ion channels by ADAMs themselves remains limited, a relatively comprehensive understanding has been achieved through *in vitro* and animal model studies investigating the effects of their major substrates, such as TNF-α, Klotho, and integrins, on cardiac ion channels. These findings create possibilities for future research on the role of ADAM family members in cardiac electrophysiology. Clinical data have shown that patients with torsades de pointes have elevated levels of the ADAM substrate IL-1 receptor, particularly in the presence of other conventional risk factors, that triggers systemic inflammation, thereby exacerbating QT interval prolongation (Düsterhöft et al., 2015; Lazzerini et al., 2017). This underscores the clinical significance of exploring the therapeutic and preventive potential of ADAMs and their substrates, including the IL-1 receptor and Klotho, in diseases associated with abnormal cardiac channel expression. Nevertheless, a major limitation is that current mechanistic investigations in this field remain largely confined to cellular or lower animal models, which fail to fully recapitulate the pathological changes that occur in the human heart and pose considerable challenges for clinical translation. Therefore, extensive future studies, including cellular and animal experiments as well as clinical investigations, are required to assess the feasibility of the clinical translation.

Pharmacological agents targeting ADAMs and their substrates at the ion channel level have demonstrated preliminary efficacy. However, most studies have focused on their roles in structural remodeling or their direct impact on pathological processes, such as myocardial inflammation. Future therapeutic approaches targeting cardiac ion channels should be systematically and rigorously developed, and given the broad effects of ADAMs in various tissues, therapeutic strategies for heart disease must be approached with caution. A deeper understanding of the precise mechanisms by which ADAMs regulate cardiac ion channels in diseases is essential for developing targeted interventions and improving the therapeutic precision and outcomes.

Recent evidence has shown that electrical remodeling caused by abnormal cardiac ion channels and structural remodeling mediated by myocardial fibrosis are not independent of each other but have complex interactions (Ramos-Mondragón et al., 2008; Zhang et al., 2015; Salvarani et al., 2017; He et al., 2019; Ahn et al., 2020). Among them, the TGF-β superfamily is the core regulator of cardiac structural remodeling. Studies have confirmed that TGF-β1 overexpression induces atrial fibrosis in mice, causes slow right atrial electrical conduction, increases left atrial conduction heterogeneity, and ultimately increases the susceptibility to AF (Verheule et al., 2004). In addition, TGF-β1 has multiple effects on electrophysiological remodeling. In neonatal rat atrial myocytes, Avila et al. found that TGF-β1 reduces the number of L-type Ca^2+^ channels by downregulating Cav1.2 mRNA expression (Ramos-Mondragón et al., 2008). Ramos-Mondragon et al. demonstrated that TGF-β1 also reduces the current density of Na^+^ channels and several K^+^ channels (Ramos-Mondragón et al., 2011). These changes may synergistically affect Ca^2+^ transients and APDs, among other factors, resulting in an increased susceptibility to arrhythmias. Studies have shown that TGF-β1 can induce membrane depolarization of myofibroblasts in neonatal rat ventricular muscle and enhance gap junction coupling between myofibroblasts and CMs, leading to membrane potential depolarization of electrically coupled CMs, which eventually leads to arrhythmogenic effects such as slow conduction and ectopic pacing (Salvarani et al., 2017). Evidence that ADAMs affect cardiac ion channel functions by regulating TGF-β is lacking. However, ADAM family members are widely involved in inflammatory responses, extracellular matrix remodeling, and profibrotic signaling regulation, and TGF-β is an important molecule connecting cardiac structural and electrical remodeling; therefore, ADAMs may be indirectly involved in ion channel remodeling and arrhythmias through TGF-β-related pathways. Clarifying this potential mechanism not only helps to improve the theoretical framework of ADAMs and their substrates in regulating cardiac ion channels but also helps to explain the mechanism by which some antifibrotic treatments improve the prognosis of arrhythmias. It provides new research directions for basic research and clinical treatments aimed at solving problems related to cardiac structural and electrical remodeling.

Overall, ADAMs and their substrates are not only important regulatory factors for cardiac inflammation and structural remodeling but may also be key hubs connecting ion channel remodeling, mitochondrial dysfunction, and profibrotic signals. An in-depth analysis of their role in cardiac electrical remodeling will help to develop new targeted intervention strategies and promote the development of precise treatments for arrhythmias.

## Glossary

ADAMs: A disintegrin and metalloproteinases
AF: Atrial fibrillation
AP: Action Potential
APD: Action Potential Duration
ARVC: Arrhythmogenic Right Ventricular Cardiomyopathy
ATP: Adenosine Triphosphate
BrS: Brugada Syndrome
**Ca** ^**2+**^: Calcium
CKD: Chronic Kidney Disease
CM: Cardiomyocyte
ECs: Endothelial Cells
EGF: Epidermal Growth Factor
ER: endoplasmic reticulum
HB-EGF: Heparin-binding Epidermal Growth Factor (EGF)-like Growth Factor
HF: Heart Failure
I/R: Ischemia-reperfusion
IFN-γ: Interferon-gamma
IL: Interleukin
IPC: Ischemic Preconditioning
IS: Indoxyl Sulfate
**K** ^**+**^: Potassium
KATP: ATP-sensitive Potassium Channels
Kir: Inwardly Rectified Potassium Channels
Kv: Voltage-gated Potassium Channels
LQTS: long QT syndrome
LTCC and TCC, respectively: L- and T-type Calcium Channels
MCU: Mitochondrial Ca^2+^ Uniporter
MI: Myocardial infarction
**mitoK**_**ATP**_: Mitochondrial KATP
mPTP: Mitochondrial Permeability Transition Pore
**Na** ^**+**^: Sodium
NCX: Na^+^/Ca^2+^ exchanger
NOX2: NADPH oxidase 2
OS: Oxidative Stress
PKCα: Protein Kinase C Alpha
ROS: Reactive Oxygen Species
RyR2: Ryanodine Receptor Type 2
**sarcK**_**ATP**_: sarcolemmal ATP-sensitive K^+^
SOCE: Store-operated Calcium Entry
SR: Sarcoplasmic Reticulum
STIM1: Stromal Interaction Molecule 1
TNF-αRANKL: Tumor Necrosis Factor-alpha
TRP: Transient Receptor Potential
TRPC6: Transient Receptor Potential Cation 6
TRPV4: Transient Receptor Potential Vanilloid 4
VGCC: Voltage-Gated Calcium Channel
VGSCs: Voltage-gated Sodium Channels
